# Addressing Suicide and Mental Health Through Universal Childhood Intervention: Results from The Seattle Social Development Project

**DOI:** 10.1007/s11121-025-01834-7

**Published:** 2025-10-18

**Authors:** Karl G. Hill, Christine M. Steeger, Marina Epstein, Jennifer A. Bailey, J. David Hawkins

**Affiliations:** 1 Institute of Behavioral Science, University of Colorado Boulder, 1440 15th St. , Boulder, CO 80309 USA; 2 Social Development Research Group, School of Social Work, University of Washington, 4320 Brooklyn Ave NE suite 700, Seattle, WA 98105 USA

**Keywords:** Childhood intervention, Elementary school, Social development, Suicide, Mental health, Raising Healthy Children

## Abstract

The objective of this study is to examine cross-over effects of the *Raising Healthy Children* universal childhood preventive intervention on adult suicide behaviors and related mental health. A nonrandomized controlled trial was conducted in elementary schools serving higher-risk areas in Seattle, Washington (during ages 6–13, grades 1–6) and followed up at ages 21–39. The panel originated in Seattle but was followed in and out of state. This study examines participants who had been in the intervention (*n* = 156) vs. control (*n* = 220) conditions in grades 1–6. The intervention provided teachers with methods of classroom management, parents with family management skills, and children with social-emotional skills training. Outcomes examined were ever suicide ideation, attempt, or completion, and DSM-IV-based criterion counts for depression, generalized anxiety disorder, PTSD, and social phobia across 6 waves, ages 21–39. At follow-up, the intervention group showed significantly lower suicide ideation and behavior, depression, generalized anxiety disorder, PTSD, and social phobia than the control group. Universal childhood preventive intervention can reduce suicide ideation and behaviors and related mental health problems in adulthood. Clinical Trials.gov ID: NCT04075019.

## Introduction

This study reports effects of a preventive intervention delivered in elementary school on adult suicidal thoughts and behavior. Suicide rates continue to increase in America, particularly for youth and young adults (Garnett & Curtin, 2023; Ruch et al., 2024). Suicide is the second highest cause of mortality among those aged 20–34, lagged only by accidents (Curtain et al., 2024). More young adults die by suicide than from cancer, birth defects, heart disease, AIDS, stroke, chronic lung disease, and pneumonia and influenza combined (Curtain et al., 2024).

### The Need for Upstream Prevention for Suicide and Mental Health Problems

A recent SAMHSA report on the National Strategy for Suicide Prevention notes an increasing interest in upstream childhood preventive efforts addressing later life suicide and related mental health problems, yet it also concludes that examinations of such cross-over effects (unanticipated beneficial effects stemming from intervention changes in shared antecedents) have been rare (Substance Abuse & Mental Health Services Administration, 2017, p. 7). Instead, the majority of interventions delivered in childhood and adolescence that address suicide have been *selective* and *indicated* interventions that target groups or individuals at elevated risk and those with early precursors for disorder who are not yet diagnosable (Institute of Medicine, 2009). For example, Kerr and colleagues (Kerr et al., 2014) examined the impact of a randomized controlled trial (RCT) of Multidimensional Treatment Foster Care (MTFC, selective/indicated) on suicide and related mental health outcomes for a sample of girls with a criminal referral who were mandated to out-of-home care. They reported that girls assigned to MTFC showed significantly greater decreases in depressive symptoms across the long-term follow-up (average 8.8 years) than the control group, and that decreases in suicidal ideation rates were slightly stronger in MTFC than in the control group, but these differences were not significant. Similarly, Sandler and colleagues (Sandler et al., 2016) reported that a family-based RCT of the *Family Bereavement Program* on a selective sample of bereaved youth ages 8–16 showed reduced suicide ideation or attempts at the 6- and 15-year follow-up assessments. That study did not report other mental health outcomes; however, in a follow-up study focusing on developmental pathways and internalizing outcomes, Sandler and colleagues reported that participants in the *Family Bereavement Program* had significantly lower odds of major depressive disorder compared to participants in the control group and marginally significant lower odds of generalized anxiety disorder (Sandler et al., 2023).

While there has been some progress in the prevention of suicide-related mental health problems with selective and indicated interventions, that approach neglects youths who are not presently identified as high risk, but who nevertheless go on to die by suicide (Wyman, 2014). Suicide prevention programming in the USA is focused almost exclusively on youth who are already suicidal and, to a lesser extent, on youth in high-risk groups defined by other symptoms or problems. Over the last decade, Wyman and others (Ayer et al., 2023; Substance Abuse & Mental Health Services Administration, 2017; Wilcox & Wyman, 2016; Wyman, 2014) have called for the inclusion of complementary universal upstream, developmental, primary prevention approaches to suicide and related mental health problems, and the *National Suicide Prevention Strategy* highlights the “need to increase the number of non-suicide-specific interventions that evaluate their impact on suicide behaviors” (Substance Abuse & Mental Health Services Administration, 2017).

Only a handful of universal primary prevention trials have examined suicide outcomes. A recent review of suicide prevention programs determined that nearly all studies in this area are focused on adults and treatment rather than prevention, and that youth suicide prevention is understudied (Mann et al., 2023). Two universal primary prevention programs have reported promising *short-term* effects (e.g., a year or less following the end of the intervention). Signs of suicide (SOS) was tested through an RCT by Aseltine and colleagues (Aseltine et al., 2007). SOS seeks to teach high school students to recognize and respond to signs of suicide in themselves and others as an emergency, much as one would react to signs of a heart attack. The authors reported significantly greater knowledge and significantly lower rates of suicide attempts among students in the intervention group, compared to the control group. In a follow-up RCT replication study, Schilling and colleagues (Schilling et al., 2016) found that, controlling for pre-test reports of lifetime attempts and recent attempts, exposure to the SOS program was associated with significantly fewer reports of suicide attempts in the 3 months following the program, but not fewer reports of planning or ideation. The SOS program has not yet reported long-term follow-up effects.

A second upstream universal preventive intervention, Familias Unidas, has also reported promising short-term effects (Vidot et al., 2016). Familias Unidas is a family-based intervention to empower Hispanic immigrant parents to build a strong parent-support network and help their adolescent children respond effectively to the risks of substance use and unsafe sexual behavior. Through an RCT, Vidot and colleagues examined suicide ideation and behavior outcomes as a growth curve at 6, 18, and 30 months post-intervention. They reported no overall significant intervention effects on suicidal behaviors. They did, however, report a promising significant interaction between treatment condition and baseline parent–adolescent communication such that intervention effects were stronger for families with initially low levels of parent–adolescent communication, indicating that the intervention may work well as a selective intervention for families with low levels of parent-adolescent communication.

Another recent evaluation of potential universal primary suicide prevention programs reported promise for the Youth Aware of Mental Health Programme (Wasserman et al., 2015). A large-scale (11,110 adolescent pupils, median age 15 years in 168 schools across 10 countries) cluster-randomized trial tested two interventions: (1) Question, Persuade, and Refer (QPR, a “gatekeeper” training module targeting teachers and other school personnel) and (2) the Youth Aware of Mental Health Programme (YAM, targeting pupils) against no treatment controls. No differences were reported at the 3-month follow-up; however, at the 12-month follow-up, YAM was associated with a significant reduction of incident suicide attempts.

Wyman and colleagues reported on the *Sources of Strength* social network-informed universal preventive intervention conducted at the high school level. While some results were promising, a test comparing the schools randomized in pairs found no significant effects (Wyman et al., 2023). However, in a follow-up cluster randomized trial in 22 schools, Wyman and colleagues report significant *Sources of Strength* program impact on reduced self-reported suicide attempts over an 18-month follow-up (Wyman et al., 2025).

To our knowledge, only one universal primary preventive intervention has reported long-term results (e.g., several years or more later) on adult suicide ideation and attempts (Wilcox et al., 2008). Wilcox and colleagues reported on the effects of the Good Behavior Game (GBG) intervention for two cohorts of elementary school (first grade) children on adult suicide ideation (Wilcox et al., 2008). GBG is a classroom behavior management game providing a strategy to help elementary teachers reduce aggressive, disruptive behavior, and other behavioral problems in children while creating a positive and effective learning environment. Results for the first cohort showed a GBG-associated reduced risk for suicide attempt at ages 19–21, although in some covariate-adjusted models the effect was not statistically robust. The impact of the GBG on suicide ideation and attempts was smaller and not significant in the replication trial involving the second cohort.

### The Raising Healthy Children Preventive Intervention

The Raising Healthy Children (RHC) preventive intervention tested in the present paper was embedded in the Seattle Social Development Project (SSDP) longitudinal study. Designed as a substance use and delinquency prevention program, RHC was implemented in grades 1 through 6 and sought to improve developmental outcomes for children in elementary school. Guided theoretically by the Social Development Model (Catalano & Hawkins, 1996; Catalano et al., 1996), RHC provided methods of management and instruction that could be used by public school teachers and adult caretakers (typically parents) to set children on a positive developmental course by promoting opportunities for children’s active involvement in classroom and family, developing children’s skills for participation, and encouraging reinforcement from teachers and parents for children’s effort and accomplishments.

Analyses examining the efficacy of RHC have identified intervention effects into adulthood. Previous tests of RHC have reported that compared to the control group, intervention participants have lower levels of school misbehavior, lifetime violence and heavy alcohol use, and higher levels of school commitment, attachment, and achievement at age 18 (Hawkins et al., 1999); better education, employment, mental health, and reduced crime at age 21 (Hawkins et al., 2005); lower lifetime prevalences of sexual intercourse, early pregnancy (or causing pregnancy), and multiple sex partners by age 21 (Lonczak et al., 2002); better socioeconomic attainment and mental health at ages 24–27 (Hawkins et al., 2008); and better health behavior, positive functioning, and adult health and socioeconomic success from ages 30–39 (Kosterman et al., 2019). To date, RHC intervention effects on suicide ideation, attempts, and associated mental health problems in adulthood have not been published.

### Purpose of Study

The present study seeks to determine whether the universal RHC preventive intervention provided in childhood had cross-over, unintended beneficial intervention effects (Ayer et al., 2023; Substance Abuse & Mental Health Services Administration, 2017) on suicide ideation and attempts and mental health outcomes in adulthood. The RHC findings reported above suggest the potential for cross-over effects on suicide ideation, attempts, and behavior and associated mental health symptomatology examined in the present study (Reider & Sims, 2016). Previous tests of the RHC intervention have reported improvements in mental health at earlier ages (Hawkins et al., 2008). The present study focuses on suicide ideation and comorbid behavior and related mental health diagnoses (Nock et al., 2012) through age 39. Although suicidal ideation and behavior have been shown to be episodic in nature and therefore difficult to detect at any single follow-up, it is important to note that most suicide deaths have been preceded by one or more prior attempts and ideation (Miller & Mazza, 2017). To capture the cumulative risk of a death by suicide, this study examines these outcomes cumulatively over an 18-year period of adulthood. Analyses address two questions. First, is the RHC universal childhood preventive intervention related to reduced suicide ideation and behaviors from age 21 to 39? Second, is the RHC universal childhood preventive intervention related to improved mental health outcomes related to suicide in adulthood?

## Method

### Sample and Intervention Assignment

Data analyses and reporting follow recommendations of the Transparent Reporting of Evaluations with Nonrandomized Designs (TREND) group (Des Jarlais et al., 2004). A CONSORT diagram describing the flow of participants is provided in a figure in the Appendix. All study measures and procedures were approved by a University Institutional Review Board. Parental permission and participant assent were obtained when participants were under age 18; consent was obtained from all participants ages 18 years and older. Data are available through a shared collaborative agreement with the current Principal Investigator of the study, and a subset is being archived at ICPSR’s National Addiction & HIV Data Archive Program (https://www.icpsr.umich.edu/web/NAHDAP/studies/39043).

This study examines adult suicide ideation and behavior and related mental health measures for individuals from the two primary intervention conditions (control and full-intervention) of a nonrandomized controlled trial of the RHC intervention. Participants were not made aware of their condition assignment and were unlikely in adulthood to remember that they had received any special parenting or teaching methods in the elementary grades. Although the schools selected for the study served relatively higher-crime neighborhoods that included children from families of relatively low socioeconomic status, the intervention package was provided universally to all students in intervention classrooms without regard to individual risk.

The first phase of the SSDP longitudinal study began in 1981 with an experimental intervention initiated with all first-grade students in eight Seattle public schools serving high-crime neighborhoods. This early intervention was randomized at both the school and classroom levels, as shown in the CONSORT diagram (appendix). In 1985, due to a change in funding, the study was expanded to a total of 18 matched Seattle public schools, adding new study conditions and additional control participants (Hawkins & Catalano, 2005). The longitudinal sample was established in 1985 from the population of 1053 students entering Grade 5 in these 18 schools, 808 (77%) of whom assented, with their parents’ consent, to participate in the longitudinal study. Thereafter, all fifth-grade students in each school received either the intervention or no intervention according to their school’s intervention assignment. This resulted in a nonrandomized controlled trial with four conditions, two of which—the full intervention and control conditions—are the focus of this report. The full intervention group consisted of those who received at least two semesters of intervention—one in Grades 1 through 4 and one in Grades 5 and 6—with an average dose of 4.13 years of intervention exposure. The control group received no intervention from the project throughout.

Other conditions did not meet these criteria and are not discussed in this report. Twenty-four of the 808 respondents moved between intervention and control schools more than once and thus could not be classified and were excluded from the analysis. Prior intervention analyses have consistently found significant intervention effects differentiating the full-intervention and control groups, but the late intervention group and the parent-training only groups were not significantly different from the control group (Hawkins et al., 1999, 2008; Kosterman et al., 2019); thus, these groups were excluded from the present analyses. This study examines outcomes for 376 youths in the two primary conditions of the RHC study: control (*n* = 220) and full intervention (*n* = 156). A study flowchart and group sample sizes at each point are provided in the CONSORT figure.

This panel was then followed into adulthood. Retention across ages 21, 24, 27, 30, 33, and 39 assessments averaged 90% retention. Nonparticipation at each assessment was not consistently related to the participant’s gender, ethnicity, or early initiation of alcohol, tobacco, marijuana, other illicit drug use, or the mental health measures examined in this study.

### Intervention Components

The full intervention consisted of three core components (more fully described in prior reports, e.g., Hawkins et al. (1999), Hawkins et al. (2005), Kosterman et al. (2019), Lonczak et al. (2002)).

- **Teacher** **staff development**: Each year, during Grades 1 through 6, teachers received 5 days of in-service training in classroom management and instructional methods, including proactive classroom management, interactive teaching, and structured cooperative learning (Abbott et al., 1998; Cummings, 1983; Cummings et al., 1982; Hawkins et al., 1988; Slavin et al., 1991).
- **Child competencies**: Additionally, first-grade teachers received instruction in the use of a cognitive and social skills training curriculum (Shure & Spivack, 1982) and, during Grade 6, a study consultant provided students with training in refusal skills (Comprehensive Health Educational Foundation, 1999). All teachers in the full intervention condition participated in the child competencies and/or staff development trainings.
- **Parent workshops**: When children were in Grades 1 through 3, parents were offered a seven-session curriculum in child behavior management skills (Hawkins & Catalano, 1987) and a four-session curriculum in skills for supporting their children’s academic development (Hawkins & Catalano, 2003b). During Grades 5 and 6, parents were offered a five-session curriculum designed to strengthen skills to reduce their children’s risks for problem behaviors (Hawkins & Catalano, 2003a). Forty-three percent of intervention parents attended at least one parenting class during Grades 1 through 3 (Hawkins & Catalano, 1987), and 29% attended at least one class during Grades 5 or 6, indicating that the parent workshop component had less reach than the teacher and child components.

## Measures

### Outcomes

The outcome measures in this study were assessed across ages 21, 24, 27, 30, 33, and 39. Repeated assessments were combined across ages to increase outcome prevalence for analyses, given the rarity of some outcomes. Any suicide ideation, attempt, or completion was coded as binary (0/1) across this age range. Related mental health measures were computed as the mean DSM-IV (Robins et al., 1999) symptom count for each outcome across this age range.

The *suicide ideation, attempts or completion* measure was assessed using three suicide-related measures across adulthood. Two items were drawn from the DSM-IV Major Depressive Episode module included in the Diagnostic Interview Schedule (Robins et al., 1999): “Do you think a lot about committing suicide?” “Have you attempted suicide?”. In addition, death certificates were obtained for each deceased study participant and coded for cause of death, and coded as “suicide” when specifically noted as such on the certificate. The suicide *ideation, attempts or completion* measure was then coded as *no* (none of these, 0) or *yes* (1, one or more of these) at each assessment age across adulthood, and the final measure was then recoded as no, never (0), or yes, at some point (1) across ages 21 to 39. Of the participants in the conditions examined in this study, 16.2% reported ever having suicidal ideation, 2.4% reported attempts, and 1.9% were identified as having completed suicide between the ages of 21 and 39. A table of suicide ideation, attempts, and completion prevelances reported or identified by each interview wave is included in the a appendix, Table 3. Overall, 18.1% of participants were coded for suicide ideation, attempts, or completion during this age range.

*Major Depressive Episode*, *Generalized Anxiety Disorder*, *Post Traumatic Stress Disorder*, and *Social Phobia* were assessed using items from the Diagnostic Interview Schedule, which was adapted to be consistent with the DSM-IV at each age. Diagnostic criterion counts were computed for each outcome at each age and then coded as the mean number of symptoms within disorder across ages 21–39. In addition, dichotomous diagnostic measures (0 *did not meet criteria*, 1 *met criteria*) for each related mental health outcome at each age and then overall (met criteria at any age 21–39) were calculated for the descriptive analyses.

*Demographic Variables*. To examine baseline equivalence and potential attrition bias, demographics were also assessed. *Gender*, *ethnicity*, and *neighborhood disorganization* (e.g., broken and abandoned buildings, neighborhood crime, etc.) were reported by the respondent. Socioeconomic status was indicated by eligibility for the federal *free lunch program*. *Childhood mobility* and whether participants were born to a *teen mother* were assessed through parent reports.

### Statistical Analyses

All participants were included in intent-to-treat analyses examining intervention impact, employed linear and logistic regression using Mplus v8.0 and full information maximum likelihood (FIML) estimation with robust standard errors (MLR) to account for missing outcome data and to address non-normality (Chou et al., 1991). Given the observed differences at baseline on having a teen mother (see Attrition and Internal Validity below), this measure was included as a covariate in all outcome analyses (Gottfredson et al., 2015).

## Results

### Baseline Equivalence and Attrition

Analyses were conducted to ascertain the initial comparability of intervention and control groups on factors that might be related to intervention condition and influence outcomes as well as whether the groups remained comparable on these factors in adult follow-up (ages 21–39 combined). Results are presented in Table 1. Differences between intervention and control groups were examined for measures that could potentially confound the outcomes examined: gender, ethnicity, socioeconomic status, mobility, and neighborhood disorganization. The groups did not differ on gender (*p* <.64), ethnicity (*p* <.84), or socioeconomic status, as measured by proportion eligible for the school lunch (*p* <.92). Given that control students were added to the study at grade 5 and the requirement that full-intervention students attended project schools for more than one semester in grades 1 through 4 and in grades 5 and 6, it is important to rule out differences in residential stability, a potential threat to internal validity. Analyses comparing the full-intervention and control groups found no significant differences in mean number of years living in Seattle by grade 6 (*p* <.27) or residence in disorganized neighborhoods at age 16 years as indicated by students’ self-reports of rundown housing, crime, poor people, drug-selling, gangs, and disorderly and undesirable neighbors in their neighborhoods (*p* <.98). The mothers of control group participants were more likely to have been teen mothers to the participants (*p* <.006). Therefore, all analyses of treatment associations control for child of a teen mother status. Additionally, a correlation matrix between the potential covariates and the outcomes is presented in the Appendix, Table 4. Among the covariates examined, childhood low socioeconomic status and neighborhood disorganization and gender were correlated with some of the outcomes examined.

**Table 1:** Characteristics of baseline measures, control, and full intervention groups at baseline and in the follow-up analysis sample

Baseline characteristics assessed	Baseline (1985)				Age 21–39 follow-up (1996–2014)			
	Control (*n* = 220)	Full (*n* = 156)	Statistic	*p*-value	Control (*n* = 214)	Full (*n* = 154)	Statistic	*p*-value
Gender (female)	46.4%	49.4%	*χ*2 = 0.219	0.640	47.2%	49.0%	*χ*2 = 0.059	0.808
Caucasian (v. other)	44.5%	46.2%	*χ*2 = 0.041	0.839	45.8%	46.4%	*χ*2 = 0.000	0.987
Percent eligible for school lunch program	56.8%	55.8%	*χ*2 = 0.009	0.923	55.7%	54.9%	*χ*2 = 0.001	0.970
Percent born to a teen mother	19.1%	8.3%	***χ***2 = 7.619	0.006	19.8%	8.5%	*χ*2 = 8.027	0.005
Mean years living in town (by age 12)	10.6	11.1	*t*(334) = − 1.10	0.271	10.6	11.1	*t*(324) = −1.15	0.253
Mean neighborhood disorganization	0.052	0.055	*t*(352) = − 0.03	0.979	0.035	0.061	*t*(343) = −0.27	0.785

Results from the attrition analysis (Table 1) showed the same pattern of significance (born to teen mom) and nonsignificance (all other included demographic variables), suggesting no major differential attrition bias. This pattern of baseline equivalence at the outset as well as after accounting for attrition by looking at the analysis sample only is consistent with all prior evaluations of this intervention (Hawkins et al., 1999, 2005, 2008; Hill et al., 2014; Kosterman et al., 2019; Lonczak et al., 2002).

### Descriptive Analyses

**Fig. 1:**
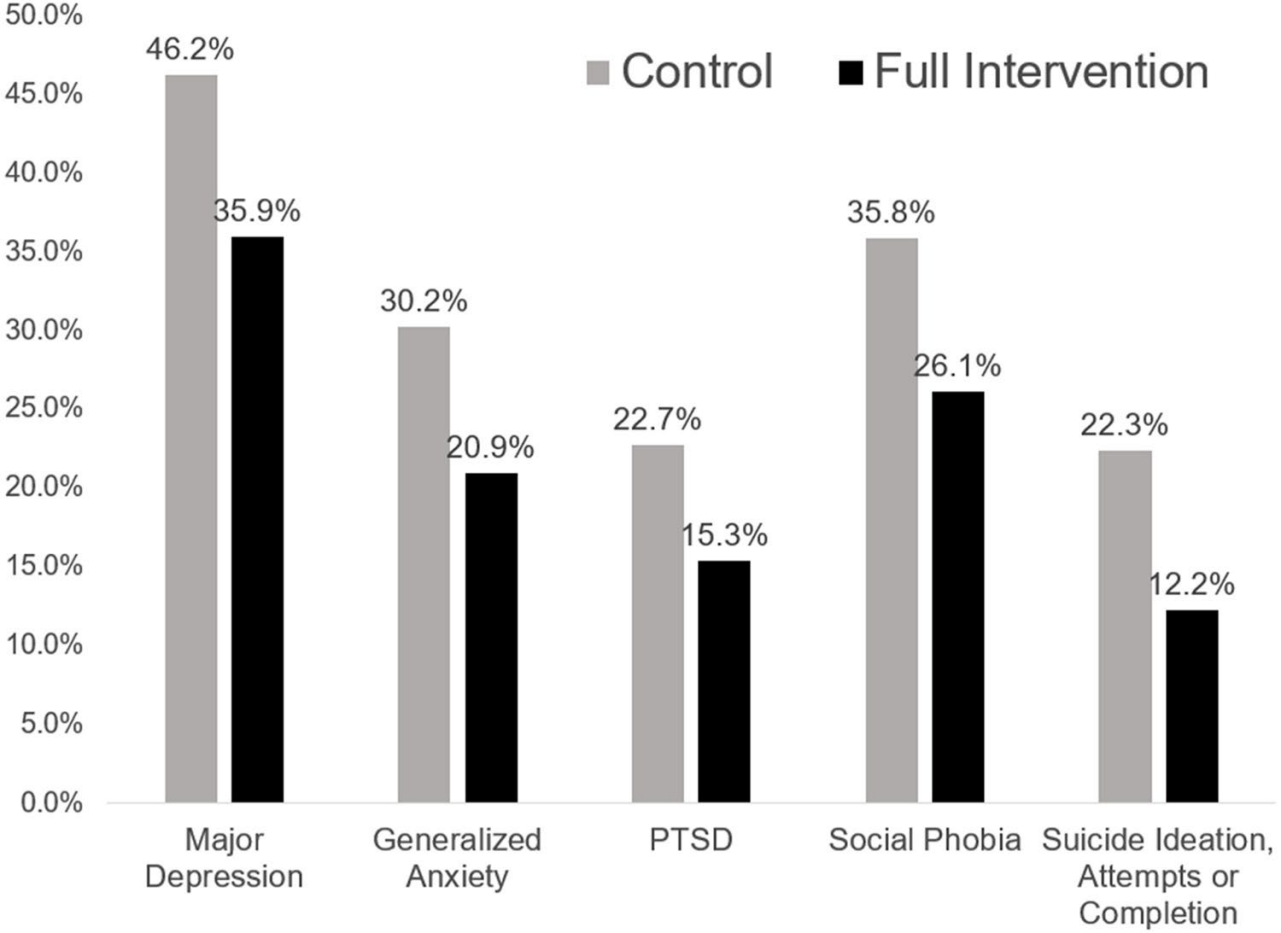
Prevalences by intervention: ever diagnosis for depression, generalized anxiety, PTSD, social phobia, and ever suicide ideation, attempts, or completion, ages 21–39

Figure 1 presents outcome prevalences across ages 21–39 for control and full-intervention groups. Prevalences of any suicide ideation, attempt, or completion and of symptoms reaching diagnostic cutoffs for each mental health condition were significantly lower in the full intervention compared to the control group.

### RHC Intervention Main Effect Outcome Analyses

Results from regression and logistic regression analysis of intervention condition predicting each outcome are presented in Table 2. Standardized estimates, standard errors, and *p*-values are presented for continuous outcomes. Logits, standard errors, adjusted odds ratios, and *p*-values are presented for the binary suicide ideation, attempts, or completion outcome. Associations significant at the *p* <.05 level or less are bolded.

**Table 2:** Regression results for intervention outcome analyses

	Outcomes ages 21–39				
	Major depression* β (SE)	Generalized Anxiety* β (SE)	PTSD* β (SE)	Social Phobia* β (SE)	Suicide ideation, attempts, or completion** Logit (SE)
RHC Intervention (control/full conditions)	− 0.270 (.10) ***p*** < 0.009	− 0.276 (.10) ***p*** < 0.008	− 0.262 (.10) ***p*** < 0.007	− 0.211 (.10) *p* < 0.051	− 0.695 (.30) 0.50 (OR) ***p*** < 0.021
Born to a teen mother (covariate)	0.092 (.13) *p* < 0.134	0.022 (.14) *p* < 0.876	− 0.020 (.06) *p* < 0.723	− 0.062 (.13) *p* < 0.631	0.274 (0.37) 1.315 (OR) *p* < 0.453

*β*, standardized beta; *SE*, standard error; *Logit*,logit estimate; *OR* = odds ratio. ^*^Mean DSM-IV symptoms across ages 21–39. ^**^Ever (0/1) across ages 21 to 39. Associations significant at the *p* <.05 level or better are in bold

Results show a positive impact of the Raising Healthy Children intervention across all outcomes. On average across ages 21–39, the intervention group compared to the control group reported fewer symptoms of major depressive episode, generalized anxiety disorder, and post-traumatic stress disorder and a lower probability of suicide ideation, attempts, or completion. The association between the intervention and social phobia symptoms was marginally significant. Effect sizes for the mental health measures were in the small-to-medium range (*β* = 0.262 to 0.276), and for the suicide outcome in the medium range (OR = 0.50).

## Discussion

The present study tested whether Raising Healthy Children (RHC), a universal preventive intervention administered in elementary school, was associated with lower rates of suicidal ideation and behaviors and comorbid mental health problems in adulthood. Results indicate a consistent pattern of beneficial effects of the RHC intervention, including lower odds of suicide ideation, attempts, or completion, as well as lower levels of depression, generalized anxiety, and PTSD through age 39, nearly three decades after the intervention ended. Levels of social phobia were lower in the full intervention group, but only marginally so. It is noteworthy that there was a consistent pattern of intervention differences across multiple related outcomes assessed across a broad period of adulthood.

A growing body of literature has identified unintended, beneficial “crossover” effects of preventive interventions for youth that focus on family management (e.g., clear rules, consistent moderate discipline), family bonding or relationship quality, child bonding to school, and child social skills (Bailey, 2009). For example, the *Familias Unidas* and *Family Checkup* programs aimed at reducing substance use, risky sex, and other adolescent problem behaviors have shown crossover effects on suicidal behaviors and mental health in adolescence and young adulthood (Connell et al., 2021; Vidot et al., 2016). Prior tests of the RHC intervention intended to reduce substance use and antisocial behavior also found crossover effects on other outcomes, including educational attainment, financial health, and mental health in adolescence and well into adulthood (Hawkins et al., 1999, 2005, 2008; Kosterman et al., 2019; Lonczak et al., 2002). In addition, crossover effects of RHC on the next generation have been observed, with offspring of individuals who received RHC showing fewer childhood developmental delays, having lower levels of externalizing behaviors, and having stronger academic and problem-solving skills in childhood and better financial functioning in young adulthood (Hill et al., 2020). This study extends prior work on crossover effects of RHC by demonstrating long-term effects on preventing or reducing suicidal behaviors and comorbid mental health conditions through age 39.

These results indicate the utility of the *Raising Healthy Children* universal preventive intervention provided in childhood for adult suicide prevention and mental health promotion and argue for its widespread implementation. Universal preventive interventions like RHC address the prevention paradox (Rose, 2001), which states that the majority of those who experience an adverse outcome (here, suicidality or mental health problems) come from a low-to-medium risk population. Thus, a narrow intervention focus on those who have already reported mental health problems or suicidal ideation would miss the majority of those who would benefit from it. Wider implementation of tested and effective universal preventive interventions would directly address the gap in suicide prevention programming identified by the 2012 US national suicide workgroup (Substance Abuse & Mental Health Services Administration, 2017) by including the large number of youths at low to moderate risk for suicidal behavior who do not currently benefit from selective and indicated interventions yet go on to consider, attempt, or complete suicide. Other studies reviewed here (Aseltine et al., 2007; Schilling et al., 2016; Vidot et al., 2016; Wasserman et al., 2015; Wyman et al., 2025) have shown promise for the impact of universal interventions on short-term outcomes. The results reported here are consistent with the hypothesis that a theory-based intervention that improved classroom management and instruction, children’s social competence, and parenting practices during the elementary grades 1–6 can have a long-lasting impact on mental health, including suicide in adulthood. These results are also consistent with the previously reported promising findings from the Good Behavior Game (Wilcox et al., 2008). Universal, upstream childhood preventive intervention can reduce suicide ideation and behaviors and related mental health outcomes in adulthood.

### Limitations and Strengths

Some limitations should be considered when interpreting results of this study. The current results reflect a long-term follow-up from a nonrandomized controlled trial and a sample originally recruited from a limited geographic area and time (the Pacific Northwest in the 1980 s). Replication on a contemporary sample, in other communities, is warranted. Testing for equivalence of intervention groups at baseline revealed only one difference (proportion born to a teen mother), which was included in statistical models. We examined those measures most appropriate and available at the 5th grade; however, other unmeasured baseline group differences may partially explain findings. Consistent with earlier intervention papers examining intervention outcomes from this study, the present study examined only full intervention (6 years of intervention grades 1–6) vs the control group (see CONSORT diagram). Other conditions were not examined in this analysis. Additionally, given the sample size examined, in order to maximize power, data on suicide ideation, attempts, and completion were combined within and across waves from 21 to 39. Ongoing efforts employing integrative data analysis and other harmonization methods to combine data across multiple datasets will be important to unpack and examine potential differences between these suicide indicators as well as examining how they unfold over the course of development. An important strength of the study is that suicide ideation, attempts, or completion was well measured through a combination of self-report and official death records. Additional strengths include the use of prospective longitudinal data, checks for internal validity, low rates of missing data, and well-validated measures of outcomes.

### Public Health Implications

A long-standing and consistently supported finding in prevention science is that identified childhood family, school, peer, individual, and community risk and protective factors predict a wide range of later developmental outcomes, and that addressing risk and protective factors through prevention has broad effects (Ayer et al., 2023; Coie et al., 1993). Findings from this study support the use of upstream, universal preventive intervention focused on identified risk and protective factors in childhood to prevent suicide later in life.

Suicide prevention research has found successes with short-term selective and indicated targeted interventions. The present study found that the universal, developmental intervention *Raising Healthy Children* had long-term effects in reducing suicide ideation and behaviors. A strong suicide prevention strategy would embed selective/indicated interventions for those at greater risk within a universal child-focused preventive intervention addressing known childhood risk and protective factors. Suicide and related mental health problems can be reduced before they start. We now have a tested and effective upstream universal preventive intervention to include in this effort.

## Figures and Tables

**Figure:**
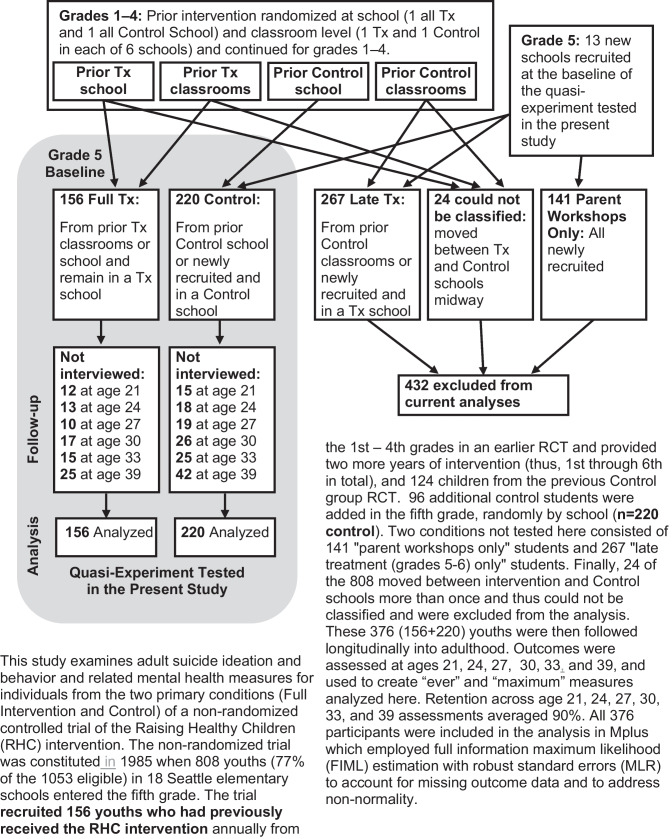
Consort diagram

## Data Availability

Data from the Seattle Social Development Project are archived at the National Addiction & HIV Data Archive Program at ICPSR (https://www.icpsr.umich.edu/web/NAHDAP/studies/39043).

## Appendix

**Table 3:** Prevalences of suicide ideation, attempts, and completion by each wave

Interview year (age in years)	Prevalences in analysis sample by each wave		
	Ideation	Attempts	Completion
1996 (21)	10.03%	1.44%	0.57%
1999 (24)	2.34%	0.99%	0.29%
2002 (27)	2.31%	0.86%	0.00%
2005 (30)	2.47%	0.67%	0.00%
2008 (33)	3.67%	0.61%	0.00%
2015 (39)	3.90%	0.97%	2.27%

**Table 4:** Correlation matrix between covariates and outcomes

		1	2	3	4	5	6	7	8	9	10	11	12
1	Gender (female)	1.00											
2	Caucasian (v. other)	− 0.04	1.00										
3	Percent eligible for school lunch program	0.05	−.040^**^	1.00									
4	Percent born to a teen mother	0.04	− 0.01	0.08	1.00								
5	Mean years living in town (by age 12)	− 0.04	0.13^*^	− 0.22^**^	0.03	1.00							
6	Mean neighh’d disorganization	− 0.02	− 0.15^**^	0.19^**^	.127^*^	0.08	1.00						
7	Tx (control vs full)	0.03	0.02	− 0.01	−.15^**^	0.06	0.00	1.00					
8	Major Depression^a^	0.08	− 0.11^*^	0.10^+^	0.05	0.01	0.17^**^	−.014^**^	1.00				
9	Generalized anxiety^a^	0.15^**^	− 0.08	0.07	0.03	− 0.04	0.24^**^	− 0.14^*^	0.70^**^	1.00			
10	PTSD^a^	0.06	− 0.03	0.05	0.00	0.01	0.21^**^	− 0.13^*^	0.48^**^	0.48^**^	1.00		
11	Social Phobia^a^	0.06	− 0.09^+^	0.12^*^	− 0.01	− 0.07	0.08	− 0.13^*^	0.40^**^	0.44^**^	0.32^**^	1.00	
12	Suicidal thoughts and behaviors^**b**^	− 0.01	0.02	0.01	0.06	− 0.02	0.12^*^	− 0.13^*^	0.50^**^	0.36^**^	0.33^**^	0.24^**^	1.00
	Mean	0.476	0.452	0.564	0.146	10.784	0.053	0.415	1.589	1.799	0.944	1.079	0.181
	Standard Dev	0.500	0.498	0.497	0.354	3.940	0.856	0.493	1.977	1.362	1.934	1.046	0.385

^a^Mean DSM-IV symptoms across ages 21–39 ^b^Ever (0/1) across ages 21–39.  + *p* < 0.10; **p* < 0.05; ***p* < 0.01, two-tailed tests
